# A Nurse-Led Education Program for Pneumoconiosis Caregivers at the Community Level

**DOI:** 10.3390/ijerph18031092

**Published:** 2021-01-26

**Authors:** Cheung Kin, Chun Yuk Jason Tsang, Lillian Weiwei Zhang, Sandy Kit Ying Chan

**Affiliations:** 1 School of Nursing, The Hong Kong Polytechnic University, Yuk Choi Road, Hung Hom, Kowloon, Hong Kong, China; lillian.zhang@polyu.edu.hk; 2 Pneumoconiosis Mutual Aid Fund, Sham Shui Po, Kowloon, Hong Kong, China; Pmaahk@gmail.com; 3 Hong Kong Buddhist Hospital, Hong Kong, China; sandychanky319@gmail.com

**Keywords:** caregivers, learning domains, mental wellbeing, nurse-led program, Orem’s self-care deficit theory

## Abstract

Pneumoconiosis is an irreversible chronic disease. With functional limitations and an inability to work, pneumoconiosis patients require support from family caregivers. However, the needs of pneumoconiosis caregivers have been neglected. This study aimed to evaluate the effectiveness of a nurse-led education program, which involved four weekly 90-min workshops led by an experienced nurse and guided by Orem’s self-care deficit theory. A single-group, repeated-measure study design was adopted. Caregivers’ mental health (Hospital Anxiety and Depression Scale, HADS, four single items for stress, worriedness, tiredness, and insufficient support), caregiving burdens (caregiving burden scale, CBS), and unmet direct support and enabling needs (Carer Support Needs Assessment Tool, CSNAT) were measured at the baseline (T0), immediately after (T1), and one month after intervention (T2); 49, 41, and 28 female participants completed the T0, T1, and T2 measurements. Mean age was 65.9 years old (SD 10.08) with a range between 37 and 85 years old. The program improved the caregivers’ mental wellbeing, and reduced their caregiving burdens and their unmet support and enabling needs, both immediately (T1) and one-month after the intervention (T2). In particular, the intervention improved the caregivers’ mental wellbeing significantly, specifically depression symptoms, stress, and tiredness immediately after the intervention; and reduced most of their unmet support needs and unmet enabling needs one-month after the intervention. This was the first nurse-led program for pneumoconiosis caregivers and should serve as a foundation for further studies to test the program with robust designs.

## 1. Introduction

Worldwide, programs to support pneumoconiosis caregivers are scarce, even though there is evidence of their mental wellbeing being affected adversely [1]. In 2012, the number of cases of pneumoconiosis rose to 727,148 in China [2]. There were 23,152 new cases in 2013, and 26,873 in 2014 [3]; 2041 cases of mesothelioma were diagnosed in China as well [4]. Furthermore, there has been an increase in the prevalence of pneumoconiosis since 2000 in Australia [5] and in the United States [5]. As a subcategory of interstitial lung disease (ILD), pneumoconiosis is untreatable and irreversible [5]. Patients with pneumoconiosis experience symptoms of breathlessness, depression, coughing, sleeping difficulty, anxiety, and fatigue [6], and functional limitations such as poor daily functioning and community living skills [1]. With functional limitations and an inability to work, pneumoconiosis patients require support from family members.

At the community level, caregivers of patients with chronic respiratory diseases, such as idiopathic pulmonary fibrosis and chronic obstructive pulmonary disease (COPD), face challenges associated with the demanding caregiving tasks of palliative care. These challenges can have adverse impacts on their own health [6,7,8], such as anxiety, depression, stress, worry, fatigue, a sense of being burdened, and insufficient support [6,7,8]. In one of the limited studies on pneumoconiosis caregivers, Tang and colleagues (2011) found that they shared similar experiences to their COPD counterparts [1]. Additionally, the caregiving burden was positively related to their depressive symptoms, but negatively correlated with the availability of family support, and pneumoconiosis patients’ daily functioning [1]. There is a need to develop interventions to improve the caregivers’ mental wellbeing, but this has been ignored in the research arena.

Studies of the support needed by COPD caregivers have identified two broad categories of unmet needs in providing palliative care: direct needs (support the caregivers may need for themselves) and enabling needs (support the caregivers may need to enable them to care for the patient) [7]. Nurses are in key positions in community settings to improve support services and skill preparation for caregivers [7].

No nurse-led palliative care program was identified for pneumoconiosis or ILD, but six nurse-led palliative care programs were identified for COPD patients and their caregivers concerning advanced care planning [9]. The authors of [9] further argued that nurse-led programs can include support and education for caregivers. Caregivers for patients with ILD reported many unmet needs for supportive care, particularly related to insufficient information and lack of psychosocial support [6]. Another qualitative study [10] found caregivers living with patients with breathlessness requested educational interventions with topics such as understanding breathlessness; managing anxiety, panic, and breathlessness; managing infection; keeping active; living positively; and knowing what to expect in the future.

However, there was a lack of evidence-based interventions for caregivers of ILD [6] and pneumoconiosis sufferers. To fill the gap, this study was done to evaluate the effectiveness of a nurse-led education program for improving pneumoconiosis caregivers’ mental wellbeing and reducing their caregiving burdens and their unmet needs for direct support and enabling.

### Theoretical Framework

This study was guided by Orem’s self-care deficit theory of nursing [11,12] to enhance the caregivers’ capacities to take care of themselves and their dependents. The term “deficit” refers to caregivers’ inadequate mental wellbeing, the burdens of caregiving, and unmet needs which require a nursing intervention to improve their self-care abilities. Orem [11,12] defined two levels of self-care: caregivers’ individual care, and their dependents’ care (i.e., the caregivers need to perform the “self-care” of the dependents, who cannot perform their own self-care). Self-care behaviors can be learned through interaction and communication with deliberate actions [11,12]. Based on the self-care ability of the individual, the nursing intervention can be classified into: wholly compensatory, partially compensatory, and supportive–educative systems (the individual can perform most of the self-care, but needs assistance with some health-related care) [11,12]. The supportive, educative system was the most appropriate nursing intervention to guide this study. In this system, the nurse can provide various types of support and education to the caregivers. Furthermore, the learning process involves three domains: cognitive, psychomotor, and affective [13]. For instance, in our study, nursing interventions could include the cognitive domain (i.e., knowledge), psychomotor domain (i.e., skills), and affective domain (i.e., emotions, attitudes, and values). Orem’s self-care deficit theory has been used to guide an intervention study on Robin sequence caregivers [14] and a systematic review of 29 studies on dementia caregivers [15].

In line with Orem’s self-care deficit theory of nursing [11,12,14,15], we hypothesized that by providing supportive, educative programs to caregivers, their psychological health and unmet support needs could be improved.

## 2. Materials and Methods

### 2.1. Design and Participants

This was a single-group, repeated-measures study, with data collected at the baseline (T0), immediately after (T1), and one month after intervention (T2). Pneumoconiosis caregivers were recruited from the community through the Pneumoconiosis Mutual Aid Association (PMAA) [16] in Hong Kong. The inclusion criteria were caregivers aged ≥18 who were taking care of family members with pneumoconiosis at home. Non-Chinese speaking caregivers were excluded from the study.

### 2.2. Data Collection

Data were collected from September 2018 to December 2019. Institutional ethical clearance (HSEARS20180902002) was obtained. After explanation of the study, participants signed the consent form; they were then assisted with filling in a 15-min questionnaire.

### 2.3. Education Program

The program consisted of four weekly 90-min interactive workshops on the topics: (1) knowing pneumoconiosis and chest percussion; (2) the aging process and lifting and transfer techniques; (3) home safety and pneumoconiosis medication, and assisted feeding techniques; and (4) infection prevention and self-care for caregivers. Seven groups of participants received the program, in seven different community centers, covering all three regions of Hong Kong. The centers were located close to where the caregivers lived. The group size ranged between 5 and 13, depending on the availability of caregivers living in the area.

The workshops were delivered by a nurse with more than 30 years of working experience in respiratory and palliative care, and conducted in multi-purpose rooms equipped with audio and video technology. Each workshop started with (1) ten minutes to recap the previous topic and answer participants’ questions; (2) forty minutes of teaching and skill demonstrations; (3) thirty minutes of hands-on practice and more demonstrations; and (4) ten minutes of answering questions. Besides, extra teaching from the nurse was provided for those who had missed a previous workshop, and they were also welcomed to attend a missing workshop with the other group.

A panel of seven experts in the fields of respiratory care, palliative care, nursing, physiotherapy, medicine, and Chinese medicine validated the teaching material, with a content validity index of 1.00.

### 2.4. Instruments

The composite questionnaire consisted of well-validated scales and was validated by a panel of four experts from nursing, medicine, and social work. The content validity index was 1.00. The following describes the four sections of the questionnaire:

#### 2.4.1. Psychological Distress

The caregivers’ psychological distress was assessed by the Hospital Anxiety and Depression Scale (HADS) [17,18,19] which contains 14 items (seven each assessing anxiety and depression). The summation scores indicate levels of anxiety and depression (8–10 mild, 11–14 moderate, and 15–21 severe) [20,21]. The score of 8 is a cut off score to represent clinically relevant indications of depression and anxiety [20]. A caregiver’s stress was assessed by one single item, “How often have you felt stress when taking care of your family members with pneumoconiosis in the past six months?” using a 5-point Likert scale (1 = never, 5 = very often). Similarly, three separate single items were used to assess whether the caregivers felt worried or tired, and had insufficient support.

#### 2.4.2. Caregiving Burden Scale (CBS)

The Chinese version of the CBS contains 20 items [1,22], using a 5-point Likert scale (1 = strongly disagree, 5 = strongly agree). The scale is to evaluate the adverse personal consequences of caregiving in relation to various caregivers’ activities or roles. The summation score was used for analysis; the higher the score, the heavier the caregiving burden. The Chinese CBS has been validated for caregivers of Alzheimer patients [22]. Its reliability was further evaluated with caregivers of pneumoconiosis patients (Cronbach’s α 0.78) [1]. Furthermore, it has been used with caregivers of patients with COPD [23] and with stroke sufferers [24]. For our study, the Cronbach’s α for the overall scale was 0.84. Permission to use the Chinese CBS was obtained from the authors [1,22].

#### 2.4.3. Carer Support Needs Assessment Tool (CSNAT)

The CSNAT was used to assess the unmet needs of caregivers [7,25]. It contains 14 items, with seven for direct support needs (i.e., needs for themselves) and seven for enabling needs (i.e., needs related to enabling them to care) [25]. A 4-point Likert scale was used (1 = no needs, 4 = far more needs). Individual items were used for the data analysis [25,26]. The CSNAT has been used on COPD caregivers [7]. For our study, the Cronbach’s α for the overall scale was 0.89. Permission to adopt the CSNAT was granted by the University of Manchester and the University of Cambridge. The traditional Chinese version was also obtained from the authors.

#### 2.4.4. Demographic Data

Caregivers’ demographic information, such as age, gender, education, comorbidities, and smoking and drinking history, were collected.

Ten extra items were asked in the T1 questionnaire to evaluate the participants’ levels of satisfaction with the education program (see details in Table 5).

### 2.5. Data Analysis

All data analyses were conducted using IBM SPSS Statistics version 25 (IBM Corp., Armonk, NY, USA). Descriptive analyses were used to present the means (Ms), standard deviations (SDs), frequencies, and percentages of the study variables, particularly for the CSNAT and program evaluation. Since most of the study variables violated the normality assumption, nonparametric statistical tests were used for the data analysis. Bivariate analyses were conducted using Spearmen correlation coefficients (rs) to measure the strengths and directions of correlations between sets of two ranked variables. To determine the effect of the program, 2-tailed Wilcoxon signed rank tests were used to determine the median changes in psychological distress, caregiving burden, and caregiver support needs between T0 and T1, T0 and T2, and T1 and T2. *p* < 0.05 was treated as significant.

## 3. Results

### 3.1. Characteristics of the Participants at Baseline (T0)

All the 49 caregivers were female with a mean age of 65.85 (SD 10.08), married (n = 47, 95.9%), with children (n = 45, 91.8%), and with education at least secondary school level (n = 30, 61.2%). Most of them were spouses of the patients, but two were daughters (Table 1).

### 3.2. Psychological Distress, Caregiver Burden, and Support Needs at Baseline (T0)

Table 2 shows the results for participants’ psychological distress, caregiver burden, and support needs. For HADS, 13 (26.53%) caregivers reported mild anxiety (8–10), 8 (16.33%) moderate anxiety (11–14), and 10 (20.41%) severe anxiety (15–21). Eight (16.32%) reported mild depression (8–10), 14 (28.57%) moderate depression (11–14), and 8 (16.32%) severe depression (15–21). They also experienced moderately high stress, worry, and tiredness, insufficient support, and certain levels of caregiving burdens and support needs.

### 3.3. Bivairiate Analyses of Study Variables at Baseline (T0)

Various significant correlations among study variables were found. For instance, CBS was correlated with anxiety (HADS) (rs = 0.673, *p* < 0.01) and depression (HADS) (rs = 0.640, *p* < 0.01) (Table 3).

### 3.4. Changes in Psychological Distress, Caregiving Burden, and Support Needs over Time

Forty-nine, forty-one, and twenty-eight participants completed the T0, T1, and T2 questionnaires, respectively. From T0 to T1, T0 to T2, and T1 to T2, the caregivers’ psychological distress and burdens reduced, and their support needs reduced, but the only significant changes were the following: (1) from T0 to T1, decreases in depression (HADS) (difference = −2.550, *p* = 0.011), stress (difference = −2.841, *p* = 0.005), and tiredness (difference = −2.403, *p* = 0.016); and (2) from T0 to T2, a decrease in insufficient support (difference = −2.160, *p* = 0.031) (Table 4).

**Figure 1:**
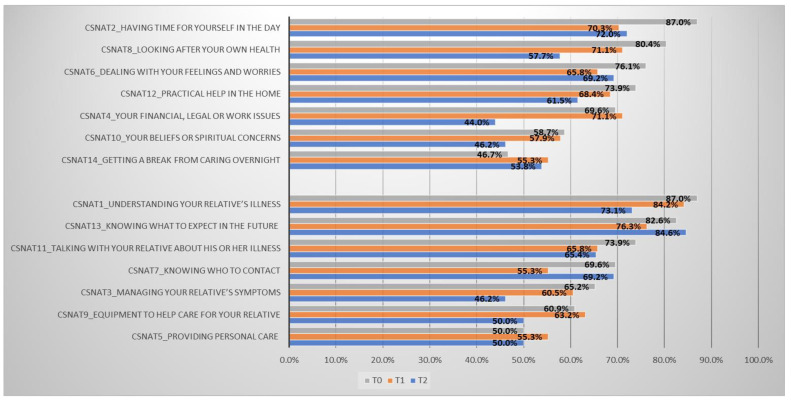
Percentages of needs, CSNAT (Carer Support Needs Assessment Tool) items, measured in T0, T1, and T2. [E] Enabling needs, CSNAT1,3,5,7,9,11,13; [S] support needs, CSNAT2,4,6,8,10,12,14.3.5. Evaluation of the Intervention.

Changes in the percentage of the needs over time for CSNAT items are presented in Figure 1. Decreasing percentages were observed for all support needs except “getting a break from caring overnight.” For enabling needs, decreasing percentages were observed for all items except “knowing what to expect in the future,” and there was no change in “providing personal care,” which ranked as the lowest need in T0.

The evaluation of the workshop quality yielded mean scores above nine (out of 10) for all items, with a mean satisfaction score of 4.95 (SD 0.22) out of 5 (Table 5).

## 4. Discussion

To summarize the main findings: 49, 41, and 28 female participants completed the T0, T1, and T2 measurements, from T0 to T1, T0 to T2, and T1 to T2, the caregivers’ psychological distress and burdens reduced, and their support needs improved.

### 4.1. Caregivers’ Characteristics and Health at Baseline

The characteristics of our caregivers were similar to those who participated in a local study conducted in 2011 [1], which was the only study in the research area. However, our caregivers were older (M 65.85, SD 10.08 vs. M 57.4, SD 11.3) and had higher caregiving burdens (M 58.2, SD 12.05 vs. M 44.3, SD 10.3). Compared with COPD caregivers, our pneumoconiosis caregivers seemed to be older, to consist of more females, and to have more psychological distress. The mean ages of the COPD caregivers ranged from 51.33 (SD 13.97, ranged from 23–82) in Jordan [21] to 62.55 (SD 13.78, ranged from 28–85) in Italy [27] and 63 (SD 12) in Hong Kong [23]. In contrast to our female-dominated sample, 61% (n = 40) and 73.75% (n = 35) of COPD caregivers were male in Jordan [21] and Italy [27] respectively.

In terms of caregiver health, COPD caregivers suffered from a mean comorbidity of 2.03 (SD 1.86, ranged from 0–7) [27]. This is comparable to our findings. However, pneumoconiosis caregivers might have higher levels of psychological distress. Using the same scale of HADS, our caregivers had an anxiety score of 9.43 (SD 5.06), which was higher than the COPD caregivers with a score of 8.39 (SD 3.55). Additionally, our caregivers had a depression score of 8.96 (SD 4.92), which was higher than the COPD caregivers, with 8.13 (SD 3.59) [21]. Similarly, our caregivers had higher caregiving burdens (CBS mean score 58.20 (SD 12.05)) than COPD caregivers (with a CBS mean score of 50 (SD 14)) [23], stroke caregivers (48.55 SD 11.38) [24], and mild dementia caregivers [22]. We found that our caregivers were overweight, with a mean BMI of 29.31 (SD 11.89). Our findings suggest that further studies should investigate the health issues of pneumoconiosis caregivers. Studies of other caregivers have found that their health can affect their ability to complete caregiving tasks and take care of themselves [28,29,30].

### 4.2. Effects of the Nurse-Led Intervention and Implications

Our study found Orem’s self-care theory-driven, nurse-led intervention for pneumoconiosis caregivers was effective in reducing their psychological distress and burdens (Table 4), and improved six out of seven (85.71%) support needs and five out of seven (71.43%) enabling needs. Reviewing our baseline assessment findings (Table 2), among 14 needs (seven support and seven enabling needs), six support needs were listed in the top seven needs. In addition, the ranking of caregivers’ support needs, in descending order was: having time for self, looking after own health, practical health at home, dealing with own feelings, financial, legal or work issues, getting a break, and beliefs and spiritual concerns. For enabling needs, the descending order was: understanding the illness, knowing what to expect in the future, talking about the illness with patient, needing equipment to help care, knowing who to contact, managing patient’s symptoms, and providing care. The increases in the percentages of two needs, “getting a break” and “knowing what to expect in the future” over time in our study might indicate that the intervention helped caregivers to identify their unmet needs. Similar findings were observed in caregivers for palliative care in the community [31]. Thus, relevant strategies should be added to the program to equip caregivers with how to take breaks from caring overnight, and where to obtain the knowledge of what to expect in the future.

The areas in which significant improvements occurred reflected the effect of the program’s content. In particular, the workshops were conducted based on Orem’s self-care deficit theory to use supportive, educative nursing interventions through interaction and communication [11,12,14]. An interactive mode was adopted, with demonstrations and return demonstrations targeting three learning domains [13]. For instance, the nurse would explain the proper use of the hands to perform chest percussion (i.e., cognitive domain), and then performed the technique on a caregiver with questions and answers, and then the caregivers would perform the action to ensure they could carry out the skill appropriately (i.e., psychomotor domain). The proper use of other equipment, such as wheelchairs, rollators, walking frames, canes, and inhalation devices (dose inhalers and aero chambers), was explained as well. Furthermore, the nurse would address the caregivers’ feelings (i.e., affective domain) in each workshop. For instance, the participants were allowed to express their negative feelings and frustrations. Understanding of the disease progression of pneumoconiosis with pragmatic coping strategies, such as relaxation exercises and mindfulness, were delineated and demonstrated. The participants were satisfied with this interactive mode of intervention (Table 5). Our promising results might also indicate that some “self-care deficits” were improved, which is in line with our hypothesis.

In addition to the theory-driven intervention, the promising results might also have been due to the appropriate dose of intervention. A current systematic review found that the more successful nurse-led programs are those with shorter durations, for example, four weekly workshops [32]. However, the review [32] also found that interventions with follow-up periods could sustain the effect. As mentioned earlier, this study was conducted through the partnership of an NGO (PMAA) and a university. The PMAA has close connections with pneumoconiosis patients and caregivers. The PMAA staff, such as social workers, could be trained by the nurse to follow up with the caregivers to ensure their compliance, and tackle the problems they encounter. Furthermore, the PMAA staff can be trained on how to use CSNAT to assess the needs of caregivers periodically, and relevant strategies can be provided to handle their unmet needs.

We observed a high attrition rate in the study. One of the main reasons for the drop out was to take care of grandchildren. Daycare services could be provided to allow caregivers to attend the workshops.

Besides, although anxiety, burdens, and worry were improved through the intervention, they were not significantly improved. One of the reasons could be due to the sensitivity of the outcome variables. For instance, burden was found to be less sensitive, and hence it is not recommended to use this solely to measure an intervention’s effect [33]. In addition, the weak associations of caregivers’ support and enabling needs with other variables (Table 3) might indicate that these needs should be assessed directly [34]. Grande and colleagues found that if these needs were addressed by an intervention, the caregiver’s health could be improved [35]. Our study found that these needs were correlated with one another. Particularly, “managing patient symptoms” and “needing equipment to help care” were significantly correlated with almost all other needs (Table 3). Nevertheless, our nurse-led intervention model can serve as a foundation for further studies of pneumoconiosis caregivers. It can also be informative for long term respiratory diseases, such as COPD.

### 4.3. Limitations

First, the design of a single-group, repeated-measures study limits its generalizability. Second, the small sample size of the study could have increased the possibility of type II error and thus yielded false-positive results [36]. Furthermore, the intervention’s effects diminished over time. Since this is one of the first studies in the area, this study will serve as a foundation for designing large-scale studies to confirm the results [36]. In addition, our study suggested a small effect size (smallest, tiredness 0.272), which indicated that a larger sample size is demanded (115 participants, effect size 0.272, significance 0.05, and power 0.80).

## 5. Conclusions

To our knowledge, this is the first nurse-led supportive, educative program, guided by Orem’s self-care deficit theory, to support pneumoconiosis caregivers in a community setting. In addition, cognitive, psychomotor, and affective learning domains were incorporated in the nursing intervention. As we hypothesized, the results of this theory-based intervention are promising—improved mental wellbeing for caregivers, reduced burdens, and fewer unmet support and enabling needs, both immediately and one-month after the intervention. Particularly, the intervention significantly reduced caregivers’ depression symptoms, stress, and tiredness immediately after the intervention; and reduced two of each of their unmet support and enabling needs one-month after the intervention.

## Figures and Tables

**Table 1:** Characteristics of participants (N = 49).

Characteristics	Number	%
Marital status	N = 49	
Single	2	4.1
Married	47	95.9
Children	N = 49	
0	4	8.2
1–2	13	26.5
3–4	29	59.2
>4	3	6.1
Education	N = 49	
No education	4	8.2
Primary school	15	30.6
Middle school	23	46.9
College (or above)	7	14.3
Comorbidities	N = 48	
Cardiovascular	15	31.3
Diabetes	4	8.3
Cardiovascular & Diabetes	9	18.8
None	9	18.8
Other *	11	22.9
	Mean ± SD (Range)	
Age (N = 47)	65.85 ± 10.08 (37–85)	
Children (N = 49)	2.80 ± 1.43 (0–7)	
Household size (N = 49)	2.29 ± 1.59 (0–5)	
BMI (N = 41)	29.31 ± 11.89 (16.74–68.61)	

* Other disease history included any kind of surgery, chronic pain (sciatica), bronchitis, and renal calculi.

**Table 2:** Characteristics of participants’ psychological distress, caregiver burden, and support needs (N = 49).

Variables	Mean ± SD (Range)	
Psychological distress		
HADS: Anxiety (N = 49; scores range 0–21)	9.43 ± 5.06 (0–20)	
HADS: Depression (N = 49; scores range 0–21)	8.96 ± 4.92 (1–19)	
Stress (N = 48; scores range 1–5)	3.65 ± 1.28 (1–5)	
Worry (N = 49; scores range 1–5)	3.63 ± 1.28 (1–5)	
Tiredness (N = 49; scores range 1–5)	3.65 ± 1.18 (1–5)	
Insufficient support (N = 48; scores range 1–5)	3.60 ± 1.32 (1–5)	
CBS (N = 46; scores range 20–100)	58.20 ± 12.05 (29–76)	
		Rank of needs
CSNAT 1 (N = 46; scores range 1–4) [E] *	2.91 ± 1.05 (1–4)	1
CSNAT 2 (N = 46) [S] ^#^	2.72 ± 1.00 (1–4)	2
CSNAT 3 (N = 46) [E]	2.26 ± 1.16 (1–4)	10
CSNAT 4 (N = 46) [S]	2.46 ± 1.17 (1–4)	6
CSNAT 5 (N = 46) [E]	2.00 ± 1.17 (1–4)	12
CSNAT 6 (N = 46) [S]	2.52 ± 1.15 (1–4)	5
CSNAT 7 (N = 46) [E]	2.28 ± 1.09 (1–4)	9
CSNAT 8 (N = 46) [S]	2.65 ± 1.10 (1–4)	3
CSNAT 9 (N = 46) [E]	2.30 ± 1.21 (1–4)	8
CSNAT 10 (N = 46) [S]	1.98 ± 1.02 (1–4)	13
CSNAT 11 (N = 46) [E]	2.43± 1.11 (1–4)	7
CSNAT 12 (N = 46) [S]	2.57 ± 1.19 (1–4)	4
CSNAT 13 (N = 46) [E]	2.65 ± 1.06 (1–4)	3
CSNAT 14 (N = 45) [S]	2.02 ± 1.23 (1–4)	11

* [E] Enabling needs, ^#^ [S] support needs.

**Table 3:** Spearman’s rho (rs) among study variables at the baseline T0 (N = 49).

	1	2	3	4	5	6	7	8	9	10	11	12	13	14	15	16	17	18	19	20	21
1	1																				
2	0.858 **	1																			
3	0.673 **	0.640 **	1																		
4	0.539 **	0.448 **	0.390 **	1																	
5	0.571 **	0.567 **	0.472 **	0.788 **	1																
6	0.672 **	0.628 **	0.555 **	0.647 **	0.704 **	1															
7	0.295 *	0.384 **	0.259	0.484 **	0.639 **	0.581 **	1														
8	0.057	0.132	0.154	0.109	0.096	−0.020	0.039	1													
9	0.151	0.009	0.118	0.063	0.080	0.102	0.114	0.278	1												
10	0.219	0.133	0.204	0.199	0.293	0.008	0.143	0.303 *	0.345 *	1											
11	0.103	−0.091	0.048	−0.072	−0.005	−0.191	0.053	0.211	0.584 **	0.550 **	1										
12	−0.011	0.048	0.025	0.007	0.100	−0.016	0.135	0.362 *	0.135	0.566 **	0.284	1									
13	0.323 *	0.253	0.299 *	0.195	0.215	0.072	0.031	0.414 **	0.227	0.632 **	0.399 **	0.509 **	1								
14	0.224	0.085	−0.116	0.050	0.138	0.024	0.115	0.226	0.148	0.255	0.261	0.040	0.371 *	1							
15	0.429 **	0.381 **	0.356 *	0.035	0.124	0.030	0.073	0.262	0.222	0.249	0.368 *	0.128	0.439 **	0.181	1						
16	0.220	0.203	0.205	0.092	0.165	−0.049	−0.068	0.228	0.483 **	0.467 **	0.543 **	0.212	0.574 **	0.094	0.365 *	1					
17	0.125	0.134	0.025	−0.048	0.002	−0.101	−0.137	0.322 *	0.242	0.516 **	0.382 **	0.462 **	0.467 **	0.113	0.289	0.662 **	1				
18	0.017	0.010	−0.005	0.129	0.204	−0.122	0.119	0.511 **	0.186	0.450 **	0.542 **	0.367 *	0.524 **	0.416 **	0.189	0.346 *	0.326 *	1			
19	0.241	0.236	0.167	−0.073	0.134	0.028	0.087	0.160	0.357 *	0.440 **	0.569 **	0.303 *	0.462 **	0.314 *	0.444 **	0.436 **	0.419 **	0.297 *	1		
20	0.101	0.078	0.163	−0.061	0.074	−0.067	0.231	0.506 **	0.333 *	0.451 **	0.514 **	0.202	0.434 **	0.287	0.413 **	0.238	0.214	0.577 **	0.435 **	1	
21	−0.009	0.020	0.023	0.121	0.235	0.116	0.353 *	0.359 *	0.410 **	0.383 **	0.538 **	0.414 **	0.441 **	0.228	0.064	0.348 *	0.320 *	0.593 **	0.424 **	0.541 **	1

Note: 1 = HADS: anxiety; 2 = HADS: depression; 3 = CBS; 4 = worrisome; 5 = stress; 6 = tiresome; 7 = insufficient support; 8 = CSNAT1; 9 = CSNAT2; 10 = CSNAT3; 11 = CSNAT4; 12 = CSNAT5; 13 = CSNAT6; 14 = CSNAT7; 15 = CSNAT8; 16 = CSNAT 9; 17 = CSNAT10; 18 = CSNAT11; 19 = CSNAT12; 20 = CSNAT13; 21 = CSNAT14 * *p* < 0.05; ** *p* < 0.01.

**Table 4:** Changes in psychological distress, caregiving burden, and support needs over time.

Study Variables	Changes			Changes			Changes		
	T1-T0 with *p*-Value ^3^			T2-T0 with *p*-Value ^3^			T2-T1 with *p*-Value ^3^		
	Z	Sig. (2-Tailed)	N	Z	Sig. (2-Tailed)	N	Z	Sig. (2-Tailed)	N
HADS: Anxiety	−1.815 ^1^	0.070	39	−0.819 ^1^	0.413	26	−0.733 ^2^	0.464	25
CBS	−0.729 ^1^	0.466	36	−0.178 ^2^	0.859	26	−0.471 ^1^	0.638	25
Worry	−1.744 ^1^	0.081	39	−0.327 ^1^	0.744	26	−0.537 ^1^	0.591	25

Note: * *p* < 0.05; ** *p* < 0.01; ^1^ based on positive ranks; ^2^ based on negative ranks; ^3^ Wilcoxon signed rank test. Bold: variables with significant results.

**Table 5:** Evaluation of the program (N = 41).

	Evaluation Items ^1^	Mean ± SD (Range)
1	Number of workshops (N = 41; scores range 1–10)	9.12 ± 1.49 (5–10)
2	Venue (N = 41; scores range 1–10)	9.46 ± 1.12 (5–10)
3	Number of participants in the workshop (N = 41; scores range 1–10)	9.37 ± 1.43 (3–10)
4	Appropriateness of the content (N = 41; scores range 1–10)	9.63 ± 0.86 (6–10)
5	Understandability of the content (N = 41; scores range 1–10)	9.76 ± 0.54 (8–10)
6	Flow of the arrangements (N = 41; scores range 1–10)	9.73 ± 0.63 (7–10)
7	Transportation (N = 31; scores range 1–10)	9.29 ± 1.62 (2–10)
8	Performance of speaker and workers (N = 41; scores range 1–10)	9.78 ± 0.52 (8–10)
9	Overall satisfaction (N = 40; scores range 1–5)	4.95 ± 0.22 (4–5)
10	Making a friend in the workshop (N = 40)	Number (%)
	Yes No	37 (92.5%) 3 (7.5%)

^1^ Note: items 1–8: using a 10-point Likert scale (10 = most satisfactory, 1 = least satisfactory); item 9: using a 5-point Likert scale (5 = most satisfactory, 1 = least satisfactory); item 10: using a dichotomous scale (with a yes or no answer).

## References

[1] Tang W.K., Yip W.C., Lum C.M., Xiang Y.T., Lee E., Ungvari G.S. (2011). Caregiving burden and quality of life of pneumoconiosis caregivers in Hong Kong. Heart Lung.

[2] Han B., Liu H., Zhai G., Wang Q., Liang J., Zhang M., Cui K., Shen F., Yi H., Li Y. (2016). Estimates and Predictions of Coal Workers’ Pneumoconiosis Cases among Redeployed Coal Workers of the Fuxin Mining Industry Group in China: A Historical Cohort Study. PLoS ONE.

[3] Wang B., Wu C., Kang L., Huang L., Pan W. (2018). What are the new challenges, goals, and tasks of occupational health in China’s Thirteenth Five-Year Plan (13th FYP) period?. J. Occup. Health.

[4] Zhao J., Zuo T., Zheng R., Zhang S., Zeng H., Xia C., Yang Z., Chen W. (2017). Epidemiology and trend analysis on malignant mesothelioma in China. Chin. J. Cancer Res..

[5] Perret J.L., Plush B., Lachapelle P., Hinks T.S., Walter C., Clarke P., Irving L., Brady P., Dharmage S.C., Stewart A. (2017). Coal mine dust lung disease in the modern era. Respirology.

[6] Lee J.Y.T., Tikellis G., Corte T.J., Goh N.S., Keir G.J., Spencer L., Sandford D., Khor Y.H., Glaspole I., Price J. (2020). The supportive care needs of people living with pulmonary fibrosis and their caregivers: A systematic review. Eur. Respir. Rev..

[7] Farquhar M. (2018). Assessing carer needs in chronic obstructive pulmonary disease. Chron. Respir. Dis..

[8] Nakken N., Janssen D.J., van den Bogaart E.H., Wouters E.F., Franssen F.M., Vercoulen J.H., Spruit M.A. (2015). Informal caregivers of patients with COPD: Home Sweet Home?. Eur. Respir. Rev..

[9] Ora L., Mannix J., Morgan L., Wilkes L. (2019). Nurse-led integration of palliative care for chronic obstructive pulmonary disease: An integrative literature review. J. Clin. Nurs..

[10] Farquhar M., Penfold C., Benson J., Lovick R., Mahadeva R., Howson S., Burkin J., Booth S., Gilligan D., Todd C. (2017). Six key Topics Informal Carers of Patients with Breathlessness in Advanced Disease want to learn about and why: MRC phase I study to inform an educational intervention. PLoS ONE.

[11] Hartweg D.L. (2013). Dorothea Orem: Self-Care Deficit Theory.

[12] Orem D.E. (1991). Nursing: Concepts of Practice.

[13] Hoque M. (2016). Three Domains of Learning: Cognitive, Affective and Psychomotor. J. EFL Educ. Res..

[14] Demoro C., Fontes C.M.B., Trettene A.D.S., Cianciarullo T.I., Lazarini I.M. (2018). Applicability of Orem: Training of caregiver of infant with Robin Sequence. Rev. Bras. Enferm..

[15] Waligora K.J., Bahouth M.N., Han H.R. (2019). The Self-Care Needs and Behaviors of Dementia Informal Caregivers: A Systematic Review. Gerontologist.

[16] Pneumoconiosis Mutual Aid Association (PMAA) Introduction of PMAA. https://pmaa.org.hk/.

[17] Zigmond A.S., Snaith R.P. (1983). The hospital anxiety and depression scale. Acta Psychiatr. Scand..

[18] Marques A., Jacome C., Cruz J., Gabriel R., Brooks D., Figueiredo D. (2015). Family-based psychosocial support and education as part of pulmonary rehabilitation in COPD: A randomized controlled trial. CHEST.

[19] Leung C.M., Wing Y.K., Kwong P.K., Lo A., Shum K. (1999). Validation of the Chinese-Cantonese version of the hospital anxiety and depression scale and comparison with the Hamilton Rating Scale of Depression. Acta Psychiatr. Scand..

[20] Hansson M., Chotai J., Nordstöm A., Bodlund O. (2009). Comparison of two self-rating scales to detect depression: HADS and PHQ-9. Br. J. Gen. Pract..

[21] Al-Gamal E., Yorke J. (2014). Perceived breathlessness and psychological distress among patients with chronic obstructive pulmonary disease and their spouses. Nurs. Health Sci..

[22] Fuh J.L., Wang S.J., Liu H.C., Wang H.C. (1999). The caregiving burden scale among Chinese caregivers of Alzheimer patients. Dement. Geriatr. Cogn. Disord..

[23] Lee E., Lum C.M., Xiang Y.T., Ungvari G.S., Tang W.K. (2010). Psychosocial condition of family caregivers of patients with chronic obstructive pulmonary disease in Hong Kong. East. Asian Arch. Psychiatry.

[24] Lau C.G., Tang W.K., Wong K.S., Mok V., Ungvari G.S. (2012). Predictors of the depressive symptomatology of the family caregivers of Chinese stroke patients in Hong Kong. J. Psychiatr. Ment. Health Nurs..

[25] Ewing G., Brundle C., Payne S., Grande G. (2013). The Carer Support Needs Assessment Tool (CSNAT) for use in palliative and end-of-life care at home: A validation study. J. Pain Symptom Manag..

[26] Alvariza A., Holm M., Benkel I., Norinder M., Ewing G., Grande G., Hakanson C., Ohlen J., Arestedt K. (2018). A person-centred approach in nursing: Validity and reliability of the Carer Support Needs Assessment Tool. Eur. J. Oncol. Nurs..

[27] Ivziku D., Clari M., Piredda M., De Marinis M.G., Matarese M. (2019). Anxiety, depression and quality of life in chronic obstructive pulmonary disease patients and caregivers: An actor-partner interdependence model analysis. Qual. Life Res..

[28] Bayly M., Morgan D., Kosteniuk J., Elliot V., Froehlich Chow A., Peacock S., McLean A., O’Connell M.E. (2019). Protocol for a systematic review on interventions for caregivers of persons with mild cognitive impairment and early dementia: Does early stage intervention improve caregiver well-being and ability to provide care?. BMJ Open.

[29] Beach S.R., Schulz R. (2017). Family Caregiver Factors Associated with Unmet Needs for Care of Older Adults. J. Am. Geriatr. Soc..

[30] Litzelman K. (2019). Caregiver Well-being and the Quality of Cancer Care. Semin. Oncol. Nurs..

[31] Aoun S.M., Grande G., Howting D., Deas K., Toye C., Troeung L., Stajduhar K., Ewing G. (2015). The impact of the carer support needs assessment tool (CSNAT) in community palliative care using a stepped wedge cluster trial. PLoS ONE.

[32] Amo-Setien F.J., Abajas-Bustillo R., Torres-Manrique B., Martin-Melon R., Sarabia-Cobo C., Molina-Mula J., Ortego-Mate C. (2019). Characteristics of nursing interventions that improve the quality of life of people with chronic diseases. A systematic review with meta-analysis. PLoS ONE.

[33] Sörensen S., Pinquart M., Duberstein P. (2002). How Effective Are Interventions with Caregivers? An Updated Meta-Analysis. Gerontologist.

[34] Sklenarova H., Krumpelmann A., Haun M.W., Friederich H.C., Huber J., Thomas M., Winkler E.C., Herzog W., Hartmann M. (2015). When do we need to care about the caregiver? Supportive care needs, anxiety, and depression among informal caregivers of patients with cancer and cancer survivors. Cancer.

[35] Grande G.E., Austin L., Ewing G., O’Leary N., Roberts C. (2017). Assessing the impact of a Carer Support Needs Assessment Tool (CSNAT) intervention in palliative home care: A stepped wedge cluster trial. BMJ Support. Palliat. Care.

[36] Hackshaw A. (2008). Small studies: Strengths and limitations. Eur. Respir. J..

